# Using Anterior Segment Optical Coherence Tomography to Monitor Disease Progression in Peripheral Ulcerative Keratitis

**DOI:** 10.1155/2018/3705753

**Published:** 2018-06-28

**Authors:** Aakriti Garg, Joaquin De Rojas, Priya Mathews, Albert Hazan, James Lin, Danielle Trief, Leejee H. Suh

**Affiliations:** Ophthalmology, Columbia University College of Physicians and Surgeons, New York, NY, USA

## Abstract

We report two cases of peripheral ulcerative keratitis (PUK) imaged with anterior segment optical coherence tomography (AS-OCT). The first patient had prolonged nonsteroidal anti-inflammatory drug use, while the second had inflammatory arthritis by laboratory findings without any systemic findings as well as possible concurrent tuberculosis. In both patients, AS-OCT demonstrated corneal thinning at the onset of the disease with improvement six months after initiation of intensive medical therapy. Our cases highlight the need for a multidisciplinary approach and careful monitoring in PUK cases, especially with objective measures such as corneal thickness assessed with AS-OCT.

## 1. Introduction

Peripheral ulcerative keratitis (PUK) is a sight-threatening disorder characterized by a crescent-shaped area of inflammation at the stromal margin associated with an epithelial defect, presence of inflammatory cells, and thinning and destruction of the stroma [1, 2].

Three million cases of PUK occur yearly [3], and approximately half of those that are noninfectious can be attributed to vasculitides [4] such as rheumatoid arthritis, polyarteritis nodosa, systemic lupus erythematosus, Sjögren's syndrome, and sarcoidosis [1]. The peripheral cornea's profound vascular supply, predominance of Langerhans cells, and high concentrations of complement and immunoglobulins provide an environment especially prone to inflammation [5, 6].

While PUK-associated thinning of the cornea can be seen on slit lamp biomicroscopy, our group sought to demonstrate these morphological changes on anterior segment optical coherence tomography (AS-OCT) (Carl Zeiss Meditec, Dublin, CA). AS-OCT is a noncontact technology that produces resolution ranging from 4 to 7 uM [7, 8], which makes AS-OCT the ideal modality for imaging corneal histology and pathology. AS-OCT has been described in studying the topography of microbial keratitis [9] and opacified Mooren's ulcers [10].

Our case series describes two PUK patients who were treated with topical and oral steroids as well as amniotic membrane transplant. Their clinical improvement was confirmed by the resolution of disease on AS-OCT.

## 2. Methods

A retrospective, noncomparative case series was performed at Columbia University Medical Center after approval by the Institutional Review Board, complied with the Health Insurance Portability and Accountability Act regulations, and adhered to the tenets of the Declaration of Helsinki. Both patients presented underwent a written informed consent process. At every clinic visit, Snellen best corrected visual acuity (BCVA), intraocular pressure (IOP), slit-lamp biomicroscopy, and AS-OCT were recorded.

## 3. Results

### 3.1. Case 1

A 73-year-old woman presented with pain, redness, and worsening vision in the right eye several months after uncomplicated cataract surgery. She had no medical history and her surgical history included Ex-PRESS glaucoma shunt of the right eye and radial keratotomy in both eyes. She was found to have postoperative cystoid macular edema (CME) and was started on topical prednisolone acetate and diclofenac, a topical nonsteroidal anti-inflammatory drug (NSAID). After being lost to follow-up for two months, she returned with a decline in BCVA of 20/20 soon after cataract surgery to 20/60. Her CME had resolved, but she now had superonasal corneal thinning in the right eye with an overlying epithelial defect that stained with fluorescein and was 4 mm vertically and 2 mm horizontally in size. She also had pigment epithelial erosions more prominent inferiorly throughout her corneas in both eyes. Her radial keratotomy incisions were intact, and central corneal tomography was stable based on Scheimpflug imaging (Pentacam, Oculus Inc., Lynnwood, WA). PUK was diagnosed; moxifloxacin, doxycycline 100 mg bid, vitamin C 1 g bid, and artificial tears were started and prednisolone and diclofenac were discontinued.

Lab workup for systemic inflammatory and infectious conditions revealed a positive QuantiFERON gold but was otherwise negative. Chest x-ray was negative. In collaboration with the patient's internist, oral prednisone was deemed safe and a moderate dose was added to the patient's treatment regimen for PUK.

**Figure 1:**
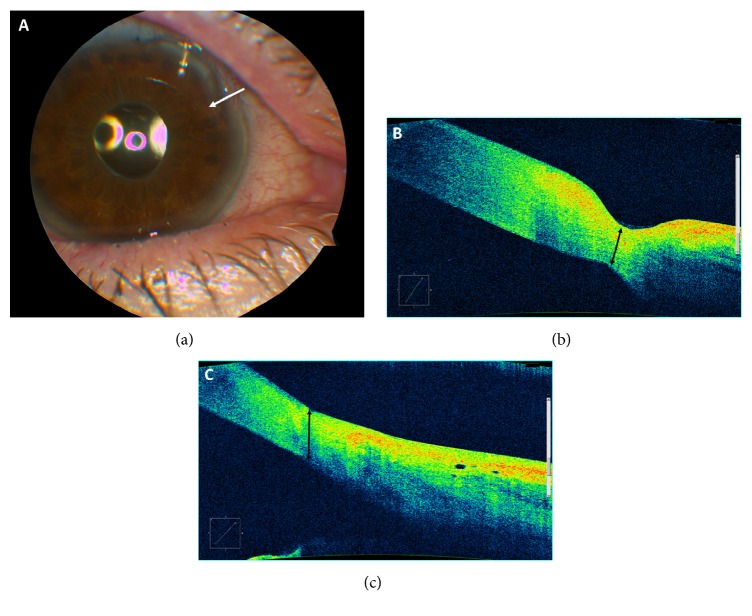
Superotemporal limbal ulcer of Patient 1.** Panel (a)**: slit-lamp examination (white arrow indicates primary area of thinning).** Panel (b)**: AS-OCT image at presentation demonstrating area of significant ulceration and thinning measured at 380 microns. The interface of the abnormal and normal cornea appears to be the most hyperreflective, which may be a sign of higher density of cellular materials in this region.** Panel (c)**: AS-OCT six months after presentation with flattening of the ulcer and subsequent improvement of thinning to 537 microns after medical therapy. The cystic region that remains to the right of the arrow may be an area of abnormal healing.

In this case, the patient's PUK was attributed to long-term use of a topical NSAID. After this was discontinued and prednisone was used for three months, she improved and has been stable with a BCVA of 20/25.  Figure 1 demonstrates a slit-lamp photograph of the case at presentation and AS-OCT images before and after treatment.

### 3.2. Case 2

A 76-year-old man was referred to our service for progressive redness and pain in the right eye. Six months earlier, his disease had been diagnosed as conjunctivitis, episcleritis, and senile furrow degeneration and he had been unsuccessfully treated with topical tobramycin/dexamethasone. His ocular and medical history and review of systems were unremarkable. BCVA in the right eye was 20/25 and slit-lamp examination revealed limbal injection, fine inferior keratic precipitates, and temporal corneal thinning with an overlying epithelial defect that measured 5 mm vertically and 2 mm horizontally and stained with fluorescein. The presumptive diagnosis of PUK was made and the patient was started on moxifloxacin q.i.d., doxycycline 100 mg bid, vitamin C 1 g bid, and topical lubricants.

Lab workup was notable for positive purified protein derivative (PPD) and QuantiFERON gold. Cyclic citrullinated peptide antibody test, a marker for rheumatoid arthritis, was also positive. All other laboratory assays were unremarkable and chest x-ray was normal. An infectious disease specialist was consulted at this time who recommended isoniazid, pyridoxine, and rifampin for the treatment of latent tuberculosis. We also consulted the rheumatology service that recommended we proceed with immunosuppression using prednisone 1 mg/kg and mycophenolic acid.

**Figure 2:**
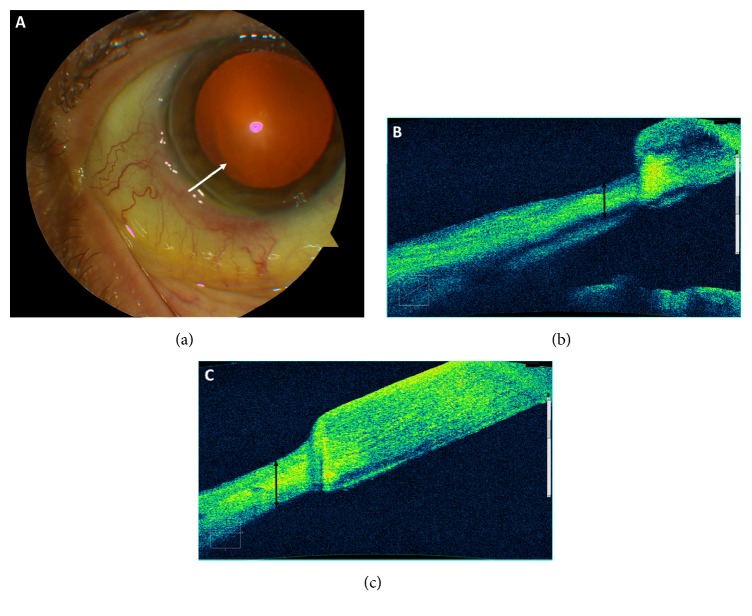
Inferotemporal limbal ulcer of Patient 2.** Panel (a)**: slit-lamp examination with white arrow drawn across primary area of thinning.** Panel (b)**: anterior segment optical coherence tomography (AS-OCT) image at presentation demonstrating area of significant corneal thinning (measured at 330 microns) and irregularity adjacent to an area of relatively normal cornea. As in Figure 1, the ulcer edges and interface between abnormal and normal cornea demonstrate hyperreflectivity.** Panel (c)**: AS-OCT six months after presentation with improvement of thinning to 450 microns after medical therapy.

His condition improved after one month and a prednisone taper was initiated. However, PUK recurred, at which point oral prednisone was restarted at the original moderate dose and amniotic membrane was placed via a PROKERA lens (Bio-Tissue, Doral, FL) to promote epithelial corneal healing. He returned two weeks later with worsening keratitis at which point the lens was removed and topical prednisolone acetate t.i.d. was initiated. Symptoms stably improved for three months, after which he was very slowly tapered off topical and systemic prednisone. At his last visit, 16 months after presentation, he remains asymptomatic with a BCVA of 20/30.  Figure 2 demonstrates a slit-lamp photograph taken at presentation and AS-OCT images taken before and after treatment. The etiology for this patient's PUK was deemed related to his autoimmune disorder with possible rheumatoid arthritis. Given the need for immunosuppression, antituberculosis medications were initiated under the care of an infectious disease specialist.

## 4. Discussion

Our case series describes two atypical PUK presentations that were monitored by AS-OCT and demonstrated disease progression and improvement.

Case 1 describes a patient without underlying systemic disease who developed corneal melt after prolonged NSAID use, which has been reported in the literature [11]. The mechanism by which NSAID can cause PUK is not well understood, but it has been postulated that accumulation of hydroperoxyeicosatetraenoic acids and leukotrienes develop from blockage of the cyclooxygenase pathway, leading to an influx of neutrophils, increased production of vascular permeability mediators, and upregulation of collagenase activity that is detrimental to the cornea [12].

Case 2 represents an example of PUK in the setting of latent tuberculosis that was safely and successfully treated with topical and oral steroids, as well as a PROKERA amniotic membrane ring. In this case, steroids were started concomitantly with antituberculosis treatment, as the patient may have been exposed to tuberculosis in the past. Amniotic membrane transplantation along with anterior chamber washout has been reported as a treatment option for severe PUK with endothelial exudates, with favorable outcomes including resolution of ulcers and stromal edema [13].

Our report describes the use of AS-OCT to successfully monitor and quantify PUK-associated corneal thinning at the limbus. The “Anterior Chamber Scan” feature was used to identify, highlight, and measure the area of most significant thinning in the anterior-posterior direction, that is, the area of interest, at the time of presentation for each patient. Thickness in the area of interest was then measured at all follow-up visits with serial OCT measurements. In both cases, corneal thickness increased over the follow-up period and improved to an average thickness in the NSAID-associated PUK eye (Case 1; 537 microns) but was still thinner than desirable in the TB-associated PUK case (Case 2; 450 microns). In addition to the precise measurement of quantitative features, qualitative elements such as increase in hyperreflectivity and diffuse increase in thickness are noted in both Case 1 and Case 2 at the abnormal-normal cornea interface. Additionally, Case 1 exhibits cystic corneal features 6 months after therapy. While this finding may be too small to detect on slit-lamp biomicroscopy, it may have significant implications on visual acuity, especially if centrally located.

AS-OCT surpasses slit-lamp biomicroscopy in the monitoring of PUK in a few important ways. First, while a cornea specialist can estimate corneal thickness by slit-lamp exam, AS-OCT allows for exact measurement of thickness in microns. AS-OCT's objective measurement of corneal thickness allows better monitoring of disease progression and treatment response. Second, AS-OCT demonstrates reflective features that may serve as a marker for disease activity. It is possible that the greater the hyperreflectivity around ulcer edges, the greater the density of the cellular material causing inflammatory activity. This may potentially be used to risk stratify patients at presentation. Lastly, because AS-OCT creates precise documentation of the corneal architecture, it serves as an important tool for communication among a team of specialists, where PUK patients often have to treat their complex disease effectively.

Given the potential systemic involvement of PUK and well-known side effects of the systemic immunomodulatory agents essential to its treatment, a collaborative effort between ophthalmologists, internists, rheumatologists, and infectious disease specialists is warranted. Furthermore, we recommend that quantitative metrics such as corneal thickness measured by AS-OCT be used more frequently by ophthalmologists and data provided to nonophthalmic physicians in order to more objectively and reliably monitor disease progression and treatment response.
